# Previous chronic symptomatic and asymptomatic cerebral hemorrhage in patients with acute ischemic stroke

**DOI:** 10.1007/s00234-018-2141-y

**Published:** 2018-11-28

**Authors:** Ge-Fei Li, Yi-Lan Wu, Shuo Wang, Yan-Hui Shi, Rong Zhao, Feng-Di Liu, Yi-Sheng Liu, Mei-Ting Zhuang, Ying Zhao, Qi Sun, Guo-Hong Cui, Jian-Ren Liu

**Affiliations:** 1 Department of Neurology, Comprehensive Stroke Center, Shanghai Ninth People’s Hospital, Shanghai Jiao Tong University School of Medicine, 639 Zhizaoju Road, Huangpu District, Shanghai, 200011 China; 2 Clinical Research Center, Shanghai Ninth People’s Hospital, Shanghai Jiao Tong University School of Medicine, Shanghai, 200011 China; 3 Department of Radiology, Shanghai Ninth People’s Hospital, Shanghai Jiao Tong University School of Medicine, Shanghai, 200011 China

**Keywords:** Ischemic stroke, Susceptibility-weighted MR imaging, Previous chronic cerebral hemorrhage

## Abstract

**Purpose:**

Identifying previous chronic cerebral hemorrhage (PCH), especially asymptomatic cases in patients with ischemic stroke, is essential for proper antithrombotic management. The study aimed to further clarify the prevalence of PCH and the associated factors in patients with acute ischemic stroke using multi-modal neuroimaging including susceptibility-weighted MR imaging (SWI).

**Methods:**

This was a retrospective cross-sectional study of 382 patients with acute ischemic stroke. All patients underwent 3.0-T MRI for cranial SWI, 1.5-T or 3.0-T conventional cranial MRI, and cranial CT. Patients found with PCH were matched 1:4 with patients without PCH. Clinical manifestation, computed tomography, conventional cranial MRI, and cranial SWI were used to determine PCH. Clinical and neuroimaging findings between the patients with symptomatic vs. asymptomatic PCH were compared.

**Results:**

Thirty-six patients (36/382, 9.4%) were determined to have had a PCH. Of these 36 patients, 17 (17/36, 47.2%, or 17/382, 4.5%) had asymptomatic PCH. Multivariable analysis showed that serum total cholesterol (OR = 0.510, 95%CI 0.312–0.832, *P* = 0.007), cerebral microbleeds (OR = 6.251, 95%CI 2.220–17.601, *P* = 0.001), and antithrombotic drugs history (OR = 3.213, 95%CI 1.018–10.145, *P* = 0.047) were independently associated with PCH. Asymptomatic PCH had similar clinical and neuroimaging characteristics with symptomatic PCH.

**Conclusion:**

PCH is not uncommon in acute ischemic stroke patients. Total serum cholesterol, cerebral microbleeds on SWI, and history of antithrombotic drugs were independently associated with PCH in patients with acute ischemic stroke. Asymptomatic PCH, which is easier to be missed and has similar characteristics with symptomatic PCH, should draw much attention.

**Electronic supplementary material:**

The online version of this article (10.1007/s00234-018-2141-y) contains supplementary material, which is available to authorized users.

## Introduction

Patients with cerebral hemorrhage frequently have indications for antithrombotic therapy. This represents a therapeutic dilemma as cerebral hemorrhage is considered a contraindication to antithrombotic medication. There were no long-term randomized studies addressing this issue. Some acute ischemic stroke patients may have had a previous chronic cerebral hemorrhage (PCH) that could recur with the use of antithrombotic therapy for secondary prevention, leading to poorer outcomes [1–7].

Susceptibility-weighted MR imaging (SWI) is more sensitive to fresh or chronic cerebral hemorrhagic lesions than conventional MRI and allows the detection of PCH, especially for asymptomatic one that was easily to be misdiagnosed [8]. Identifying PCH in patients with acute ischemic stroke could be essential for prudent initiation of antithrombotic therapy, esp., intensive antithrombotic therapy. However, the prevalence of PCH and proper method to detect asymptomatic or symptomatic PCH needs to be further clarified for acute ischemic stroke patients. Therefore, this study used SWI examination, combined with multi-modal neuroimaging examinations and detailed clinical data, to observe the prevalence of PCH (symptomatic or not) and analyze the associated factors of PCH in patients admitted for acute ischemic stroke in a comprehensive stroke center.

## Methods

### Patients

This retrospective cross-sectional study analyzed patients who were diagnosed with acute ischemic stroke and admitted at the stroke center of our hospital, one of national comprehensive stroke centers between 12/12/2011 and 3/6/2013. This study was approved by the ethic committee of our hospital. Informed consent was waived by the committee because of the retrospective nature of the study. The patient inclusion criteria are presented in the Supplemental Material.

### Neuroimaging

All patients underwent CT in the emergency room to exclude acute cerebral hemorrhage. Conventional MRI and diffusion-weighted imaging (DWI) were completed within 5 days of admission. SWI was performed within 10 days of admission. Examinations were blindly reviewed by two experienced neurologists. A third senior physician was invited for consensus in case of controversy between the first two physicians. Details about the MRI examinations are provided in the Supplemental Material.

Cranial SWI were used to observe and analyze cerebral microbleeds (CMBs) [8, 9]. According to the Microbleed Anatomical Rating Scale (MARS) [10], CMB was defined as circular or oval low signal lesions with clear boundary on SWI sequence, with a diameter of 2–10 mm, while excluding lesions with similar imaging manifestations (including calcification, ferruginous sediment, cavernous hemangioma.) by combining with phase diagrams, mIP, other MRI sequences [11, 12], and cranial CT. The amount of CMBs was recorded according to the MARS for quantitative evaluation [10].

The presence of PCH was determined based on non-enhanced cranial CT, conventional cranial MRI scanning (routine scanning sequence), cranial SWI examination, and disease history. PCH referred to hypointensity on SWI magnitude images, with a diameter > 1 cm. They appeared as softened hypodense lesions on cranial CT, hypointensity on T1-weighted MRI, and hyperintensity on T2-weighted images, and on MRI (T1- or T2-weighted MRI, or FLAIR MRI) there would be a hypointense rim around the lesion, indicating iron deposition after absorption of the hematoma. In addition, cerebral hemorrhage could be differentiated from calcification using SWI filtered phase image; in filtered phase images for a left-handed system, calcification has low signal intensity, while cerebral hemorrhage has high signal intensity [11, 12]. Previous neuroimaging studies were checked as possible for patients with a medical history of cerebral hemorrhage. Besides cranial CT scan, all MRI sequences (DWI, T1-weighted MRI, T2-weighted MRI, FLAIR, and SWI) were carefully reviewed to further exclude acute intracerebral hemorrhages, and those suspected transformation hemorrhage related to the acute ischemic stroke on cranial MRI would not be defined as PCH.

### Classification of acute stroke

Stroke was classified according to the TOAST (Trial of org10172 in Acute Stroke Treatment) classification [13] and combined with disease history and imaging characteristics of the patients, and acute ischemic stroke was classified into five types: large-artery atherosclerosis (LAA), small-artery occlusion (SAA), cardiogenic embolism (CE), stroke of other demonstrated etiology (SOE), and stroke of other undemonstrated etiology (SUE). Hemorrhagic transformation during acute stroke was defined as the development of a hemorrhage after an acute ischemic stroke.

### Statistical analysis

Continuous data was expressed as mean ± standard deviation or median (range), and analyzed with the Student *t* test or the Wilcoxon rank sum test, as appropriate. Categorical data was expressed as frequencies and percentages and analyzed with the Pearson chi-square test or Fisher exact test, as appropriate. Subsequently, each variable was introduced into a logistic regression model. Factors with *P* values < 0.05 in univariable analyses were entered into the multivariable logistic regression model, screened using the backward method, where the inclusion criterion was *P* < 0.05. Statistical analyses were performed using SPSS 19.0 (IBM, Armonk, NY, USA). Two-sided *P* values < 0.05 were considered statistically significant.

## Results

Thirty-six patients (36/382, 9.4%) had PCH (Supplementary Table S1). Of these, 17 (17/36, 47.2%, or 17/382, 4.5%) had asymptomatic PCH. Multivariable analysis showed that serum total cholesterol (OR = 0.510, 95%CI 0.312–0.832, *P* = 0.007), cerebral microbleeds (OR = 6.251, 95%CI 2.220–17.601, *P* = 0.001), and antithrombotic drugs history (OR = 3.213, 95%CI 1.018–10.145, *P* = 0.047) were independently associated with PCH (Tables 1 and 2). Asymptomatic PCH had similar demographic, neuroimaging, and biochemical characteristics with symptomatic PCH (Supplementary Table S2).

**Table 1:** Univariable logistic regression of factors associated with cerebral hemorrhage

	Odds ratio	95% confidence interval	*P*
TOAST classification			
LAA vs. SUE	1.011	0.248–4.117	0.988
SAA vs. SUE	0.383	0.121–1.214	0.103
CE vs. SUE	–	–	–
SOE vs. SUE	1.386	0.470–3.517	0.625
Hypertension history	1.843	0.664–5.119	0.241
Hypertension classification	1.233	0.887–1.713	0.212
SBP	1.000	0.983–1.018	0.969
DBP	0.998	0.966–1.032	0.927
Diabetes	1.415	0.675–2.967	0.358
Fasting blood glucose	1.039	0.901–1.196	0.600
HbA1c	1.164	0.969–1.400	0.105
LDL	0.511	0.313–0.836	0.008
HDL	0.623	0.155–2.507	0.505
TC	0.498	0.318–0.778	0.002
TG	1.006	0.616–1.642	0.980
Uric acid	1.003	0.999–1.007	0.100
Creatinine	1.011	0.997–1.025	0.133
Microbleeds	7.000	2.742–17.867	<0.001
Microbleeds score	1.062	1.006–1.121	0.030
Hemorrhagic transformation	0.265	0.034–2.078	0.206
Prominent vessel sign on SWI	3.086	0.841–11.328	0.089
History of antithrombotic drugs	3.143	1.176–8.400	0.022
Brain white matter degeneration score	1.357	1.090–1.690	0.006

*TOAST* Trial of Org10172 in Acute Stroke Treatment, *LAA* large-artery atherosclerosis, *SAA* small-artery occlusion, *CE* cardio-embolism, *SOE* stroke of other demonstrated etiology, *SUE* stroke of other undemonstrated etiology, *SBP* systolic blood pressure, *DBP* diastolic blood pressure, *LDL* low-density lipoprotein, *HDL* high-density lipoprotein, *TC* total cholesterol, *TG* triglycerides

**Table 2:** Multivariable regression analysis of factors associated with cerebral hemorrhage

	Odds ratio	95% confidence interval	*P*
Total cholesterol	0.510	0.312–0.832	0.007
Cerebral microbleeds	6.251	2.220–17.601	0.001
Use history of antithrombotic drugs	3.213	1.018–10.145	0.047

Factors with *P* values < 0.05 in univariable analyses were tested using a backward method

## Discussion

The study aimed to analyze the prevalence of PCH in patients with acute ischemic stroke using SWI and to analyze the factors associated with PCH in those patients. Thirty-six (9.4%) patients were found to have PCH. Multivariable analysis results showed that TC, existence of CMBs on SWI, and medication history of antithrombotic drugs were independently associated with the presence of a PCH in patients with ischemic stroke. There were no differences in clinical characteristics between previous symptomatic and asymptomatic PCH.

In the present study, the frequency of PCH was 9.4%, or 4.5% for asymptomatic PCH, which was slightly lower than in previous studies [14, 15]. The discrepancies could be due to the selection of the subjects and geographical regions, as well as to different imaging protocols.

Patients with ischemic stroke should promptly receive antithrombotic therapy for secondary prevention if they do not have any significant contraindication, but for those with both acute ischemic stroke and PCH, symptomatic or not, long-term aggressive antithrombotic therapy should be used with caution after considering the risks and benefits to avoid re-bleeding [3–7]. There were no long-term randomized studies addressing this issue. There is a marked paucity of evidence to guide clinicians when planning the long-term management of patients with cerebral hemorrhage and cogent indications for antithrombotic therapy. Recently, a randomized controlled trial, “The REstart or STop Antithrombotics Randomised Trial (RESTART) after stroke due to intracerebral haemorrhage,” has been registered to compare starting versus avoiding antiplatelet drugs for adults surviving antithrombotic-associated ICH at 122 hospital sites in the UK [16].

CMB can be associated with poor prognosis of stroke [17, 18]. A recent study reported in White and Chinese patients with ≥ 5 CMBs withholding antiplatelet drugs during the first year after transient ischemic attack/ischemic stroke may be inappropriate; however, the risk of ICH may outweigh any benefit thereafter [19].

The present study found that there were more patients suffering from CMBs in patients with PCH compared with patients without PCH, and CMBs independently associated with existence of PCH. Thus, for patients with ischemic stroke, it could be necessary to perform SWI examination to promptly discover CMBs, as well as PCH, in order to guide antithrombotic medication.

The proper management of patients with acute ischemic stroke and PCH remains controversial [3–7, 20, 21]. Our study found the PCH was not uncommon in acute ischemic stroke patients and antithrombotic medication should be used with more caution after considering risk and benefit. The present study also suggests that there were no differences in clinical characteristics between symptomatic and asymptomatic PCH, making the latter easy to be missed, which could influence the outcomes of antithrombotic therapy and increase the risk of re-bleeding. The present study suggests that SWI could be a proper imaging modality for identifying patients with asymptomatic PCH, hence guiding the therapeutic strategy and patient management.

The limitations are presented as Supplemental Material.

## Conclusion

In this study of patients admitted in national stroke centers for acute ischemic stroke and who underwent conventional MRI and SWI and cranial CT, 9.4% were found to have a PCH. Total cholesterol, the presence of CMBs on SWI, and history of antithrombotic drugs were independently associated with the presence of PCH in patients with acute ischemic stroke. Asymptomatic PCH, which is easier to be missed but has similar characteristics of demographic, neuroimaging, and serum biochemical testing with symptomatic PCH, should draw much attention. Hence, implementing routine cranial SWI examination could show PCH more sensitively, allowing the optimal management of these patents in order to prevent re-bleeding and worsening of the clinical outcomes.

## Electronic supplementary material

ESM 1 (DOCX 32 kb)
