# Supplementary material for: Previous chronic symptomatic and asymptomatic cerebral hemorrhage in patients with acute ischemic stroke
Source: Neuroradiology. 2018 Nov 28;61(1):103–7. doi: 10.1007/s00234-018-2141-y (PMC6336746; doi:10.1007/s00234-018-2141-y)
Supplement: Supplementary file 1 — (DOCX 32 kb) [file 234_2018_2141_MOESM1_ESM.docx]

**METHODS**

**Inclusion criteria**

The inclusion criteria were: 1) confirmed diagnosis of acute ischemic stroke based on detailed clinical and imaging data; 2) underwent cranial CT, conventional cranial MRI, and SWI, in the emergency room or during hospitalization; 3) appropriate image quality (i.e. no artifact and appropriate for diagnosis); and 4) cranial CT did not show recent cerebral hemorrhagic lesion (i.e., without high density lesion). The exclusion criteria were: 1) previous history of cerebral ischemic stroke; 2) intracranial tumors, hydrocephalus, inflammatory diseases, demyelinating diseases, or neurodegenerative diseases; or 3) incomplete clinical, biochemical, or neuroimaging data.

**MRI parameters**

For SWI: TR 28.0 ms, TE 20.0 ms, slice thickness (ST) 1.5 mm, and FOV 240×220；phase images, mIP images, magnitude images were reconstructed. For 1.5-T MRI, the parameters were: 1) T1WI: TR 1800-2000 ms, TE 9-12 ms, and FOV 240×220; 2) T2WI: TR 4000 ms, TE 94 ms, and FOV 240×220; 3) FLAIR: TR 7500-8000 ms, TE 105-120 ms, and FOV 240×220; and 4) DWI: TR 5000 ms, TE 78 ms, and FOV 240×240. For 3.0-T MRI, the parameters were: 1) T1WI: TR 1800 ms, TE 8.6 ms, and FOV 240×220; 2) T2WI: TR 4000 ms, TE 104 ms, and FOV 240×220 3) FLAIR: TR 8000 ms, TE 90 ms, and FOV 240×220; and 4) DWI: TR 6000-8000 ms, TE 100 ms, b 1000, and FOV 240×220. CT was performed using a 64 row scanner.

Cranial susceptibility-weighted MRI sequences were used to observe and analyze cerebral hemorrhage and CMBs. According to the microbleed anatomical rating scale (MARS) [[1](#_ENREF_1)], CMB was defined as circular or oval low-signal lesions with clear boundary on SWI sequence, with a diameter of 2-10 mm, while excluding lesions with similar imaging manifestations (including calcification, ferruginous sediment, cavernous hemangioma, etc.) by combining with phase diagrams, mIP [[2](#_ENREF_2)], other MRI sequences, and cranial CT. The amount of CMBs was recorded according to the MARS for quantitative evaluation [[1](#_ENREF_1)].

Cranial T2 FLAIR MRI sequences were used to analyze white matter lesions. According to the Fazekas’s classification [[3](#_ENREF_3)], paraventricular and deep white matter lesions were separately graded, and their scores were summed up to get a total score (0-6 points). Paraventricular high signal: 0: no lesion; 1: cap-shaped, pencil-like thin lesions; 2: smooth halo; and 3: irregular lesions extending to deep white matter. Deep white matter high signal: 0: no lesion; 1: dotted lesion; 2: lesions started to fuse; and 3: a large area of fusion of lesions.

The presence of previous chronic cerebral hemorrhage (PCH) was determined based on non-enhanced cranial CT, conventional cranial MRI scanning (routine scanning sequence), cranial SWI examination, and disease history. PCT referred to hypointensity on SWI magnitude images, with a diameter >1 cm. They appeared as softened hypodense lesions on cranial CT, hypointensity on T1-weighted MRI, and hyperintensity on T2-weighted images, and on MRI (either T1- or T2- weighted MRI) there would be a hypointense rim around the lesion, indicating iron deposition after absorption of the hematoma. In addition, cerebral hemorrhage could be differentiated from calcification using SWI filtered phase image [[4](#_ENREF_4)]. In filtered phase images for a left-handed system, calcification has low signal intensity, while cerebral hemorrhage has high signal intensity [[2](#_ENREF_2)]. Previous neuroimaging studies were checked as possible for patients with a medical history of cerebral hemorrhage.

**LIMITATIONS**

This study has some limitations. First, this was a retrospective study. There may be some deficiency in data collection and original recording in the charts. In addition, follow-up was inconsistent, preventing for now any study on the prognosis of these patients. We did not re-evaluate SWI and neurological functions in these patients, and thus we were not able to further analyze lesion progression by SWI. In addition, due to the low incidence of the disease, we only included a small number of patients with PCH, thus limiting the generalizability of the results. Because of the Chinese medical and health insurance systems and extreme large volume of patients, all examinations (CT, conventional MRI, and SWI) could not be performed on the same day. They were nevertheless completed within 10 days of admission, but differences in timing could lead to some bias. In addition, since SWI was not performed in acute stage but within 10 days of admission, it was not used to guide the thrombolytic therapy. Nevertheless, it could eventually be used to guide the long-term antithrombotic therapy. Finally, because of conditions such as diabetes and atrial fibrillation prior to stroke, some patients were exposed to antithrombotics before their stroke, preventing reliable long-term outcome analysis. Additional studies are necessary to address these issues, preferentially with increased sample size, in order to clarify the clinical value of SWI examination in patients with acute stroke and clarify the effect of antithrombotic therapy on incidence of re-bleeding during long term follow-up in stroke patients with PCH.

**Supplementary Table S1.** Baseline characteristics according to the presence of previous chronic cerebral hemorrhage (PCH)

|  | PCH  (n=36) | Without PCH  (n=144) | *P* |
| --- | --- | --- | --- |
| Gender, n (%) |  |  | 1.000 |
| Male | 20 (55.6%) | 80 (55.6%) |  |
| Female | 16 (44.4%) | 64 (44.4%) |  |
| Age (years) | 71.9±11.4 | 71.4±12.6 | 0.929 |
| TOAST classification, n (%) |  |  | 0.121 |
| LAA | 7 (19.4%) | 60 (41.7%) |  |
| SAA | 18 (50.0%) | 46 (31.9%) |  |
| CE | 7 (19.4%) | 23 (16.0%) |  |
| SOE | 0 (0%) | 2 (1.4%) |  |
| SUE | 4 (11.1%) | 13 (9.0%) |  |
| Hypertension history |  |  | 0.235 |
| Yes | 31 (86.1%) | 111 (77.1%) |  |
| No | 5 (13.9%) | 33 (22.9%) |  |
| Hypertension classification |  |  | 0.625 |
| 0 | 5 (13.9%) | 33 (22.9%) |  |
| 1 | 3 (8.3%) | 15 (10.4%) |  |
| 2 | 9 (25.0%) | 31 (21.5%) |  |
| 3 | 19 (52.8%) | 65 (45.1%) |  |
| SBP (mmHg) | 143.3±22.1 | 143.1±21.6 | 0.853 |
| DBP (mmHg) | 82.7±11.8 | 82.9±11.6 | 0.900 |
| Diabetes history |  |  | 0.356 |
| Yes | 16 (44.4%) | 52 (36.1%) |  |
| No | 20 (55.6%) | 92 (63.9%) |  |
| Fasting blood glucose (mmol/L) | 6.79±3.00 | 6.55±2.35 | 0.712 |
| HbA1c (%) | 7.3±3.9 | 6.3±1.8 | 0.549 |
| LDL (mmol/L) | 2.52±0.67 | 2.99±0.94 | 0.006 |
| HDL (mmol/L) | 1.06±0.26 | 1.10±0.28 | 0.537 |
| TC (mmol/L) | 4.03±0.83 | 4.69±1.13 | 0.003 |
| TG (mmol/L) | 1.50±0.85 | 1.49±0.75 | 0.678 |
| Uric acid (µmol/L) | 336.6±129.1 | 300.9±95.7 | 0.096 |
| Creatinine (µmol/L) | 97.9±37.3 | 89.7±22.8 | 0.534 |
| Microbleeds on SWI |  |  | <0.001 |
| Yes | 30 (83.3%) | 60 (41.7%) |  |
| No | 6 (16.7%) | 84 (58.3%) |  |
| Microbleeds score | 4.92±6.00 | 2.34±5.70 | <0.001 |
| Hemorrhagic transformation |  |  | 0.176 |
| Yes | 1 (3.0%) | 15 (10.6%) |  |
| No | 32 (97.0%) | 127 (89.4%) |  |
| Prominent vessel sign on SWI |  |  | 0.076 |
| Yes | 4 (13.8%) | 7 (4.9%) |  |
| No | 25 (86.2%) | 135 (95.1%) |  |
| Previous use of antithrombotic drugs |  |  | 0.018 |
| Yes | 8 (22.2%) | 12 (8.3%) |  |
| No | 28 (77.8%) | 132 (91.7%) |  |
| Brain white matter degeneration score | 4.1±1.6  4 (2,6) | 3.2±1.8  3 (0,6) | 0.004 |

TOAST: Trial of Org10172 in Acute Stroke Treatment; LAA: large-artery atherosclerosis; SAA: small-artery occlusion; CE: cardio-embolism; SOE: stroke of other demonstrated etiology; SUE: stroke of other undemonstrated etiology; SBP: systolic blood pressure; DBP: diastolic blood pressure; LDL: low-density lipoprotein; HDL: high-density lipoprotein; TC: total cholesterol; TG: triglycerides; SWI: susceptibility-weighted imaging.

**Supplementary Table S2.** Comparison of the characteristics between patients with symptomatic and asymptomatic previous chronic cerebral hemorrhage (PCH)

|  | Asymptomatic  (n=17) | Symptomatic  (n=19) | P |
| --- | --- | --- | --- |
| Gender, n (%) |  |  |  |
| Male | 10 (58.8%) | 10 (52.6%) | 0.709 |
| Female | 7 (41.2%) | 9 (47.4%) |  |
| Age (years) | 71.35±11.03  74 (50,85) | 72.32±12.02  77 (50,93) | 0.805 |
| TOAST classification, n (%) |  |  |  |
| LAA | 3 (17.6%) | 4 (21.1%) | 0.672 |
| SAA | 10 (58.8%) | 8 (42.1%) |  |
| CE | 2 (11.8%) | 5 (26.3%) |  |
| SUE | 2 (11.8%) | 2 (10.5%) |  |
| Hypertension history |  |  |  |
| Yes | 16 (94.1%) | 15 (78.9%) | 0.406 |
| No | 1 (5.9%) | 4 (21.1%) |  |
| Hypertension Classification |  |  |  |
| 0 | 1 (5.9%) | 4 (21.1%) | 0.533 |
| 1 | 2 (11.8%) | 1 (5.3%) |  |
| 2 | 4 (23.5%) | 5 (26.3%) |  |
| 3 | 10 (58.8%) | 9 (47.4%) |  |
| SBP (mmHg) | 146.25±25.53  145 (110,200) | 140.47±18.61  140 (100,160) | 0.461 |
| DBP (mmHg) | 85.19±13.11  80 (70,108) | 80.35±10.27  80 (60,100) | 0.246 |
| Diabetes history |  |  |  |
| Yes | 7 (41.2%) | 9 (47.4%) | 0.709 |
| No | 10 (58.8%) | 10 (52.6%) |  |
| Fasting blood glucose (mmol/L) | 6.83±2.29  6 (4.5,11.9) | 6.76±3.62  5.65 (4.1,20.1) | 0.943 |
| HbA1c (%) | 7.79±4.84  6.15 (3.9,22.8) | 6.70±2.04  6.55 (4.6,10.5) | 0.513 |
| LDL (mmol/L) | 2.51±0.71  2.36 (1.59,3.64) | 2.53±0.65  2.55 (1.13,3.32) | 0.916 |
| HDL (mmol/L) | 1.01±0.28  0.99 (0.56,1.69) | 1.11±0.24  1.11 (0.63,1.77) | 0.286 |
| TC (mmol/L) | 4.04±0.92  4.11 (2.45,5.57) | 4.03±0.74  4.01 (2.76,5.41) | 0.984 |
| TG (mmol/L) | 1.66±0.91  1.39 (0.78,3.92) | 1.33±0.77  1.13 (0.49,3.57) | 0.255 |
| Uric acid (µmol/L) | 370.43±129.16  340.5 (202,636) | 302.79±124.36  257 (153,575) | 0.170 |
| Creatinine (µmol/L) | 99.47±39.83  89 (54,227) | 96.29±35.91  86 (62,199) | 0.621 |
| Microbleeds on SWI |  |  |  |
| Yes | 14 (82.4%) | 16 (84.2%) | 0.881 |
| No | 3 (17.6%) | 3 (15.8%) |  |
| Microbleeds score | 4.82±7.38  3 (0,28) | 5.00±4.64  4 (0,14) | 0.415 |
| History of antithrombotic therapy |  |  |  |
| Yes | 4 (23.5%) | 4 (21.1%) | 0.858 |
| No | 13 (76.5%) | 15 (78.9%) |  |
| Brain white matter degeneration score | 4.00±1.54  4 (2,6) | 4.22±1.67  4 (2,6) | 0.732 |

TOAST: Trial of Org10172 in Acute Stroke Treatment; LAA: large-artery atherosclerosis; SAA: small-artery occlusion; CE: cardio-embolism; SOE: stroke of other demonstrated etiology; SUE: stroke of other undemonstrated etiology; SBP: systolic blood pressure; DBP: diastolic blood pressure; LDL: low-density lipoprotein; HDL: high-density lipoprotein; TC: total cholesterol; TG: triglycerides; SWI: susceptibility-weighted imaging.

**REFERENCES**

1. Gregoire SM, Chaudhary UJ, Brown MM, Yousry TA, Kallis C, Jager HR, Werring DJ (2009) The Microbleed Anatomical Rating Scale (MARS): reliability of a tool to map brain microbleeds. Neurology 73 (21):1759-1766. doi:10.1212/WNL.0b013e3181c34a7d

2. Ayaz M, Boikov AS, Haacke EM, Kido DK, Kirsch WM (2010) Imaging cerebral microbleeds using susceptibility weighted imaging: one step toward detecting vascular dementia. J Magn Reson Imaging 31 (1):142-148. doi:10.1002/jmri.22001

3. Fazekas F, Chawluk JB, Alavi A, Hurtig HI, Zimmerman RA (1987) MR signal abnormalities at 1.5 T in Alzheimer's dementia and normal aging. AJR Am J Roentgenol 149 (2):351-356. doi:10.2214/ajr.149.2.351

4. Haacke EM, Mittal S, Wu Z, Neelavalli J, Cheng YC (2009) Susceptibility-weighted imaging: technical aspects and clinical applications, part 1. AJNR Am J Neuroradiol 30 (1):19-30. doi:10.3174/ajnr.A1400
